# Insights into the Ecological Diversification of the Hymenochaetales based on Comparative Genomics and Phylogenomics With an Emphasis on *Coltricia*

**DOI:** 10.1093/gbe/evad136

**Published:** 2023-07-27

**Authors:** Heng Zhao, Yu-Cheng Dai, Fang Wu, Xiao-Yong Liu, Sundy Maurice, Konstantin V Krutovsky, Igor N Pavlov, Daniel L Lindner, Francis M Martin, Yuan Yuan

**Affiliations:** Institute of Microbiology, School of Ecology and Nature Conservation, Beijing Forestry University, Beijing, China; Institute of Microbiology, School of Ecology and Nature Conservation, Beijing Forestry University, Beijing, China; Institute of Microbiology, School of Ecology and Nature Conservation, Beijing Forestry University, Beijing, China; College of Life Sciences, Shandong Normal University, Jinan, China; Section for Genetics and Evolutionary Biology (EVOGENE), Department of Biosciences, University of Oslo, Oslo, Norway; Department of Forest Genetics and Forest Tree Breeding, Georg-August University of Göttingen, Göttingen, Germany; Center for Integrated Breeding Research, George-August University of Göttingen, Göttingen, Germany; Laboratory of Population Genetics, N. I. Vavilov Institute of General Genetics, Russian Academy of Sciences, Moscow, Russia; Laboratory of Forest Genomics, Department of Genomics and Bioinformatics, Genome Research and Education Center, Institute of Fundamental Biology and Biotechnology, Siberian Federal University, Krasnoyarsk, Russia; Scientific and Methodological Center, G. F. Morozov Voronezh State University of Forestry and Technologies, Voronezh, Russia; Mycology and Plant Pathology, V.N. Sukachev Institute of Forest SB RAS, Krasnoyarsk, Russia; Department of Chemical Technology of Wood and Biotechnology, Reshetnev Siberian State University of Science and Technology, Krasnoyarsk, Russia; Center for Forest Mycology Research, Madison, Wisconsin, USA; Université de Lorraine, INRAE, UMR Interactions Arbres/Microorganismes, Centre INRAE-GrandEst-Nancy, Champenoux, France; Institute of Microbiology, School of Ecology and Nature Conservation, Beijing Forestry University, Beijing, China

**Keywords:** Hymenochaetaceae, white rot, genomics, *Coltricia*, ecological diversity, transposable elements

## Abstract

To elucidate the genomic traits of ecological diversification in the Hymenochaetales, we sequenced 15 new genomes, with attention to ectomycorrhizal (EcM) *Coltricia* species. Together with published data, 32 genomes, including 31 Hymenochaetales and one outgroup, were comparatively analyzed in total. Compared with those of parasitic and saprophytic members, EcM species have significantly reduced number of plant cell wall degrading enzyme genes, and expanded transposable elements, genome sizes, small secreted proteins, and secreted proteases. EcM species still retain some of secreted carbohydrate-active enzymes (CAZymes) and have lost the key secreted CAZymes to degrade lignin and cellulose, while possess a strong capacity to degrade a microbial cell wall containing chitin and peptidoglycan. There were no significant differences in secreted CAZymes between fungi growing on gymnosperms and angiosperms, suggesting that the secreted CAZymes in the Hymenochaetales evolved before differentiation of host trees into gymnosperms and angiosperms. Nevertheless, parasitic and saprophytic species of the Hymenochaetales are very similar in many genome features, which reflect their close phylogenetic relationships both being white rot fungi. Phylogenomic and molecular clock analyses showed that the EcM genus *Coltricia* formed a clade located at the base of the Hymenochaetaceae and divergence time later than saprophytic species. And *Coltricia* remains one to two genes of AA2 family. These indicate that the ancestors of *Coltricia* appear to have originated from saprophytic ancestor with the ability to cause a white rot. This study provides new genomic data for EcM species and insights into the ecological diversification within the Hymenochaetales based on comparative genomics and phylogenomics analyses.

**Significance**

The genetics and evolution of fungi have always been of great concern. In this study, we used 31 genomes of Hymenochaetales, including 15 newly sequenced, to study the evolution of different ecological types (ectomycorrhizal, parasitic, and saprophytic) at the genome level. The results showed that compared with those of parasitic and saprophytic species, plant cell wall degrading enzyme genes were significantly decreased in ectomycorrhizal species, while transposable elements, genome size, small secretory proteins, and secretory proteases were increased. Phylogenomic analysis showed that the ectomycorrhizal species of Hymenochaetales may have originated from saprophytic ancestors. Our study enriches the genomic resource for Hymenochaetales as well as provides fundamental views on the ecological diversification of Hymenochaetales fungi.

## Introduction

Fungi are distributed worldwide in all ecosystems. It has been estimated that 2.2–3.8 species exist but only 140,000 have been described (Hawksworth and Lücking 2017; Naranjo-Ortiz and Gabaldón 2019; Wang et al. 2019). In forest ecosystems, fungi, such as wood decomposers, soil or litter saprotrophs, and ectomycorrhizal species, play a crucial role in the fluxes of nutrients, especially carbon (Heimann and Reichstein 2008; Floudas et al. 2012; Wu et al. 2022a). Among them, wood-decomposing fungi are classified as white rot and brown rot according to their decay modes (Krah et al. 2018). Furthermore, it has been inferred that ectomycorrhizal (EcM) form a symbiotic association with about 60% of trees on earth (Steidinger et al. 2019), mainly with species of Pinaceae, Fagaceae, Betulaceae, and Myrtaceae, which are widely distributed in various forest ecosystems (Whitham et al. 2008).

The Hymenochaetales, the core group of wood-inhabiting fungi, is a species-rich order in the Agaricomycetes, Basidiomycota, consisting of more than 1,100 species with a worldwide distribution (https://www.catalogueoflife.org; accessed on November 14, 2022). Species of Hymenochaetales are primarily saprophytic causing a white rot decay (Wu et al. 2022b). Some, however, exhibit different life modes as pathogens, including important tree pathogens like *Porodaedalea*, *Onnia*, *Coniferiporia*, and *Sanghuangporus* (Wu et al. 2019, 2022b; Zhao et al. 2022b), or ectomycorrhizal symbionts, especially the genus *Coltricia* (Tedersoo et al. 2007; Wu et al. 2022b). Danielson (1984) identified the *Coltricia perennis* was EcM associates of pine. Tedersoo et al. (2007) morphologically illustrated four species of *Coltricia* formed ectomycorrhizae with *Vateriopsis seychellarum*, *Intsia bijuga*, and *Eucalyptus robusta*. And Korotkin et al. (2018) proved four samples of the genus *Coltricia* as EcM. With the genus *Coltriciella* treated as a synonym of *Coltricia*, 35 species were accepted, while many species grown on the ground of forests or decayed wood and lack of detailed studies in their lifestyle (Bian et al. 2022; Wu et al. 2022b). In addition, the genome of *Coltricia* has not been sequenced and explaining the symbiotic evolution.

With the reduction of genome sequencing costs and the rapidly increasing numbers of fungal genome data, more studies have focused on the innovation and evolution of fungal life modes using omics data analyses (Floudas et al. 2012; Sipos et al. 2017; Naranjo-Ortiz and Gabaldón 2019; Lebreton et al. 2021; Lofgren et al. 2021; Sun et al. 2022; Wu et al. 2022c). Comparative genomic analyses have suggested that white rot fungi differ from brown rot fungi by their CAZymes repertoire (Floudas et al. 2012). Furthermore, glycoside hydrolase (GH6 and GH7 families) and lytic polysaccharide monooxygenase (AA9 family) genes are more abundant in white rot fungi than in brown rot fungi, and class II lignin-modifying POD (AA2 family) are usually totally lost in brown rot fungi (Floudas et al. 2012; Kohler et al. 2015; Krah et al. 2018). Moreover, ectomycorrhizal fungi, such as some species of Boletales and Russulales, have less plant cell wall degrading enzymes (PCWDEs) compared with those of ancestral wood decomposers, as well as many lineage-specific genes concerned with the degradation of soil organic material (Kohler et al. 2015; Lebreton et al. 2021; Lofgren et al. 2021; Wu et al. 2022c). However, only a few Hymenochaetales genomes have been published until now, for instance only 18 genomes are currently available in the NCBI database (https://www.ncbi.nlm.nih.gov/genome, accessed on December 7, 2022). The few genomics studies on Hymenochaetales mostly addressed their pathogenicity, phylogeny, mitochondrial genomes, and medicinal value (Floudas et al. 2012; Chung et al. 2017; Lee et al. 2019; Caballero et al. 2020; Jiang et al. 2021; Zhao et al. 2022a), while their genomic features underlying different ecological types, including ectomycorrhizal, parasitic, and saprophytic, remain underexplored.

Here, we reveal the genome traits of ectomycorrhizal, parasitic, and saprophytic species within the Hymenochaetales using comparative genomics, mainly focusing on the secreted proteins and PCWDEs repertoires, as well as a reconstruction of their phylogenomic relationships and divergence time based on single-copy orthologous genes.

## Results

### Genome Features

**Fig. 1:**
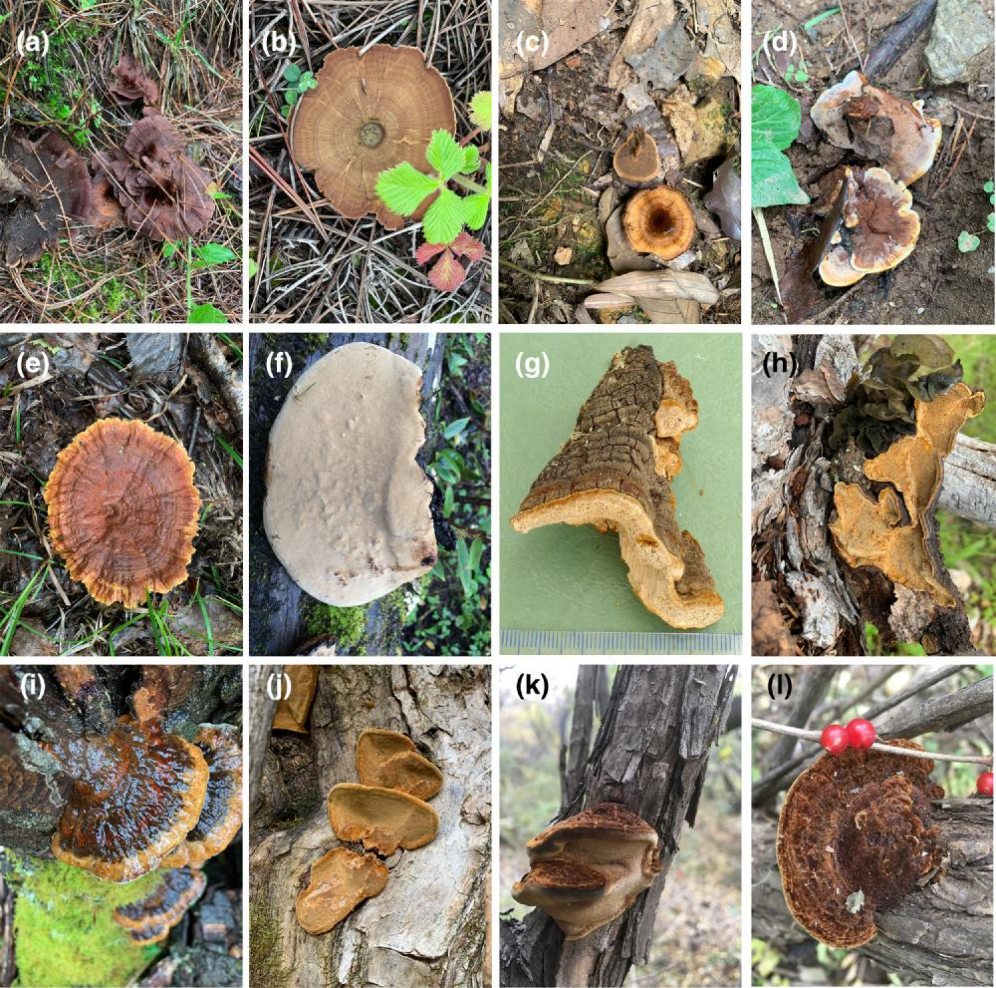
Basidiomata of Hymenochaetales. (*a*) *Coltricia abieticola*. (*b*) *Coltricia perennis*. (*c*) *Coltricia weii*. (*d*) *Onnia himalayana* (Zhao et al. 2022b). (*e*) *Onnia tomentosa*. (*f*) *Phellinus monticola*. (*g*) *Porodaedalea occidentiamericana* (Wu et al. 2022b). (*h*) *Porodaedalea qilianensis*. (*i*) *Pseudoinonotus tibeticus*. (*j*) *Sanghuangporus alpinus*. (*k* and *l*) *Sanghuangporus lonicericola*.

**Fig. 2:**
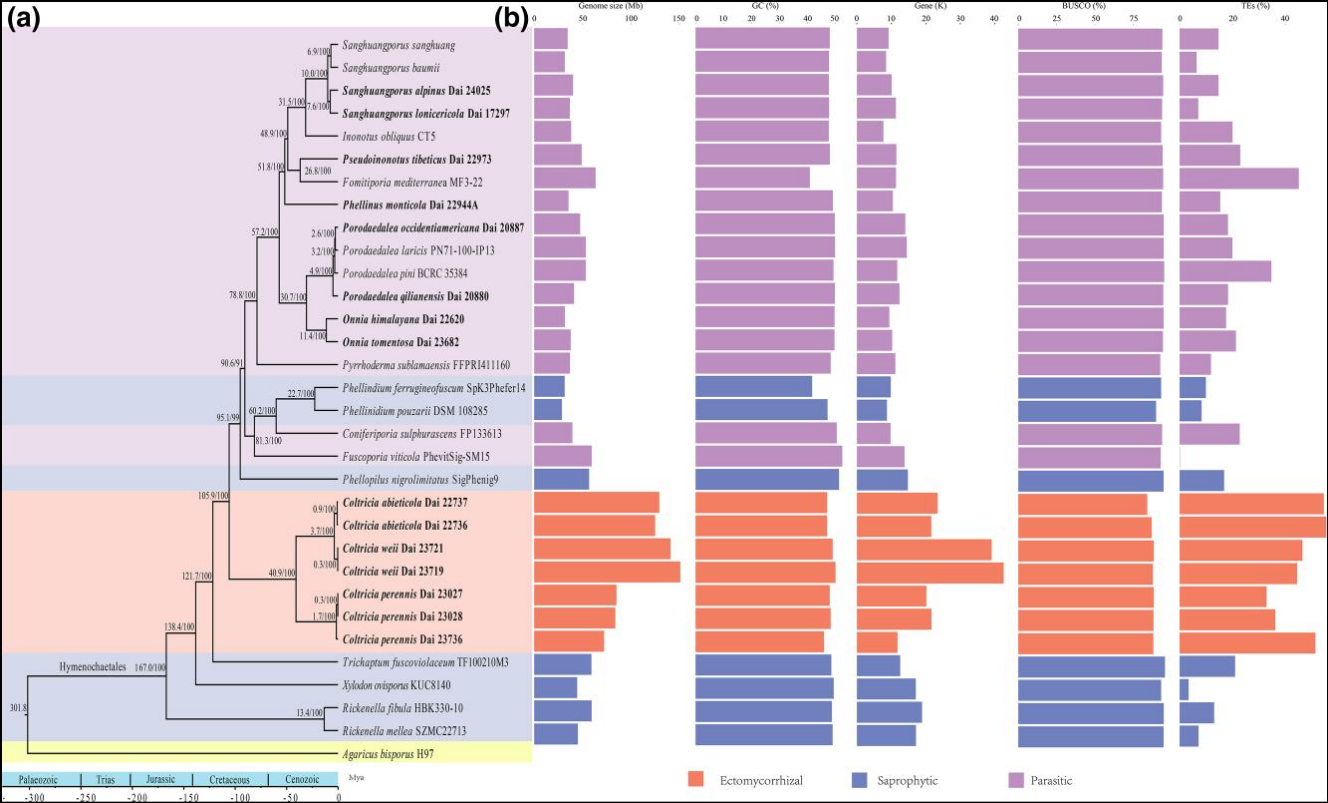
The phylogenomic relationships and genome features of 31 members of Hymenochaetales with *Agaricus bisporus* as the outgroup. (*a*) A MCC phylogenomic tree based on the 145 orthologous protein clusters of the 32 genomes with ML bootstrap values (>90%) presented at the branches nodes. (*b*) Genome features: value of genome size (Mb), GC content (%), the number of protein-coding gene models (thousands), BUSCO, and TEs in the entire genomes. The 15 new genomes generated in this study are highlighted in bold.

In the present study, 15 genomes of 11 species within Hymenochaetaceae, that is seven *Coltricia*, two *Onnia*, one *Phellinus*, two *Porodaedalea*, one *Pseudoinonotus*, and two *Sanghuangporus*, were newly sequenced and assembled (table 1, figs. 1 and 2 and supplementary File S1, Supplementary Material online).

**Table 1:** Taxonomic Affiliation, Genomic Features of 32 Genomes Used in This Study

Species name	Sample no.	Ecology	Host	Genome size (Mb)	Total gene	Total scaffolds	N50 (bp)	L50	GC (%)	BUSCO (%)	References
*Agaricus bisporus*	H97			30.2	10,606	29	262,535	35	46.5		Morin et al. (2012)
***Coltricia abieticola***	**Dai 22736**	**Ectomycorrhizal**	**Gym**	**124**.**9**	**21,590**	**24,984**	**9,898**	**2,837**	**47**.**03**	**87**.**0**	**This study**
***Coltricia abieticola***	**Dai 22737**	**Ectomycorrhizal**	**Gym**	**129**.**2**	**23,425**	**29,018**	**10,379**	**2,972**	**47**.**02**	**84**.**1**	**This study**
***Coltricia perennis***	**Dai 23027**	**Ectomycorrhizal**	**Gym**	**85**.**0**	**20,148**	**20,097**	**8,182**	**2,126**	**47**.**93**	**88**.**2**	**This study**
***Coltricia perennis***	**Dai 23028**	**Ectomycorrhizal**	**Gym**	**83**.**8**	**21,625**	**21,661**	**7,865**	**2,215**	**48**.**26**	**88**.**4**	**This study**
***Coltricia perennis***	**Dai 23736**	**Ectomycorrhizal**	**Gym**	**72**.**2**	**11,778**	**10,952**	**12,360**	**1,386**	**45**.**88**	**88**.**1**	**This study**
***Coltricia weii***	**Dai 23719**	**Ectomycorrhizal**	**Ang**	**150**.**9**	**42,579**	**41,768**	**10,182**	**3,054**	**49**.**98**	**87**.**8**	**This study**
***Coltricia weii***	**Dai 23721**	**Ectomycorrhizal**	**Ang**	**140**.**8**	**39,102**	**36,447**	**10,383**	**2,677**	**49**.**49**	**88**.**4**	**This study**
*Coniferiporia sulphurascens*	FP133613	Parasitic to saprophytic	Gym	39.4	9,756	31	19,39760	8	50.45	93.8	Chung et al. (2017)
*Fomitiporia mediterranea*	MF3-22	Parasitic	Ang	63.4	11,330	1,412	4,291,492	6	40.83	94.5	Floudas et al. (2012)
*Fuscoporia viticola* (*Phellinus viticola*)	PhevitSig-SM15	Parasitic to saprophytic	Gym & Ang	59.4	13,824	296	414,969	35	52.43	92.9	Sundy Maurice et al. (unpublished)
*Inonotus obliquus*	CT5	Parasitic	Ang	38.1	7,690	31	1,971,511	7	47.60	93.2	Duan et al. (2022)
***Onnia himalayana***	**Dai 22620**	**Parasitic**	**Gym**	**31**.**7**	**9,402**	**1,932**	**65,132**	**131**	**49**.**65**	**94**.**8**	**This study**
***Onnia tomentosa***	**Dai 23682**	**Parasitic**	**Gym**	**37**.**7**	**10,231**	**2,303**	**39,363**	**253**	**49**.**63**	**94**.**4**	**This study**
*Phellinidium pouzarii*	DSM 108285	Saprophytic	Gym	28.6	8,701	1,764	41,383	204	47.15	89.9	GenBank: SGPK00000000
*Phellinidium ferrugineofuscum* (*Phellinus ferrugineofuscus*)	SpK3Phefer14	Saprophytic	Gym	36.9	11,139	661	283,736	37	48.26	92.6	Sundy Maurice et al. (unpublished)
*Phellopilus nigrolimitatus*		Saprophytic	Gym	56.7	14,792	696	344,575	35	51.26	94.8	Sønstebø et al. (2022)
***Phellinus monticola***	**Dai 22944A**	Parasitic to saprophytic	**Ang**	**35**.**4**	**10,443**	**4,263**	**66,117**	**122**	**49**.**08**	**93**.**7**	**This study**
*Porodaedalea laricis* (*P. niemelaei*)	PN71-100-IP13	Parasitic	Gym	53.3	14,467	951	143,259	91	49.83	94.9	Pavlov, Krutovsky et al. (unpublished)
***Porodaedalea occidentiamericanna***	**Dai 20887**	**Parasitic**	**Gym**	**47**.**4**	**14,038**	**5,849**	**50,859**	**204**	**49**.**76**	**94**.**9**	**This study**
*Porodaedalea pini*		Parasitic	Gym	53.3	11,698	220	569,242	25	49.27	95.2	Chung et al. (2017)
***Porodaedalea qilianensis***	**Dai 20880**	**Parasitic**	**Gym**	**41**.**1**	**12,345**	**4,083**	**67,971**	**150**	**49**.**8**	**94**.**6**	**This study**
***Pseudoinonotus tibeticus***	**Dai 22973**	**Parasitic**	**Gym**	**49**.**0**	**11,436**	**7,100**	**20,569**	**572**	**47**.**99**	**94**.**2**	**This study**
*Pyrrhoderma sublamaensis*	FFPRI411160	Parasitic	Ang	31.4	9,820	12	3,409718	4	41.64	93.2	Chung et al. (2017)
*Rickenella fibula*	HBK330-10	Saprophytic	Bry	59.3	18,897	528	404,684	35	48.69	94.9	Korotkin et al. (2018)
*Rickenella mellea*		Saprophytic	Bry	45.2	17,134	848	361,966	27	48.96	94.8	Krizsán et al. (2019)
***Sanghuangporus alpinus***	**Dai 24025**	**Parasitic**	**Ang**	**40**.**1**	**10,081**	**5,121**	**23,522**	**459**	**47**.**62**	**94**.**5**	**This study**
*Sanghuangporus baumii*		Parasitic	Ang	31.6	8,455	217	267,109	34	47.70	93.6	GenBank: LNZH00000000
***Sanghuangporus lonicericola***	**Dai 17297**	**Parasitic**	**Ang**	**36**.**8**	**11,307**	**3,658**	**78,191**	**106**	**47**.**72**	**93**.**8**	**This study**
*Sanghuangporus sanghuang*		Parasitic	Ang	34.5	9,192	37	2,512,777	6	47.96	94.0	Jiang et al. (2021)
*Xylodon ovisporus*	KUC8140	Saprophytic	Ang	44.4	17,089	1,291	121,466	103	49.36	93.2	Min et al. (2015), Fernández-López et al. (2018)
*Trichaptum fuscoviolaceum*		Saprophytic	Gym	59.1	12,552	12	2,702,940	8	48.5	95.7	GenBank: CAJSYZ000000000

Note.—Genomes generated in this study are highlighted in bold.

Gym, gymnosperms; Ang, angiosperms; Bry, bryophyte.

In the genus *Coltricia*, the assembled genome sizes ranged from 72.2 Mb (*C. perennis* Dai 23736) to 150.9 Mb (*Coltricia weii* Dai 23719) with a GC content of 45.88% and 49.98%, respectively for Dai 23736 and Dai 23719. Between 11,778 and 42,579 predicted protein-coding gene models were predicted, respectively, which showed a rapidly evolving genome size and gene number.

In the genus *Onnia*, the assembled genome size of *Onnia himalayana* Dai 22620 was 31.7 Mb, and of *Onnia tomentosa* Dai 23682, 37.7 Mb, with a GC content of 49.65% and 49.63%, respectively, and 9,402 and 10,231 protein-coding gene models were predicted, respectively.

The assembled genome size of *Phellinus monticola* Dai 22944A was 35.4 Mb with a GC content of 49.08% and 10,443 protein-coding gene models were predicted.

In the genus *Porodaedalea*, the assembled genome size of *Porodaedalea occidentiamericana* Dai 20887 was 47.4 Mb with GC content of 49.76% and of *Porodaedalea qilianensis* Dai 20880, 41.1 Mb with GC content 49.8%; 14,038 and 12,345 protein-coding gene models were predicted, respectively.

The assembled genome size of *Pseudoinonotus tibeticus* Dai 22973 was 49.0 Mb with a GC content of 47.99% and 11,436 protein-coding gene models were predicted.

In the genus *Sanghuangporus*, the assembled genome size of *Sanghuangporus alpinus* Dai 24025 was 40.1 Mb with GC content of 47.62% and of *Sanghuangporus lonicericola* Dai 17297, 36.8 Mb with GC content of 47.72%; 10,081 and 11,307 protein-coding gene models were predicted, respectively.

From 84.1% to 94.9% of Benchmarking Universal Single-Copy Orthologs (BUSCO) were obtained in 15 genomes of Hymenochaetaceae. More genome features are listed in table 1 and supplementary File S1, Supplementary Material online.

**Fig. 3:**
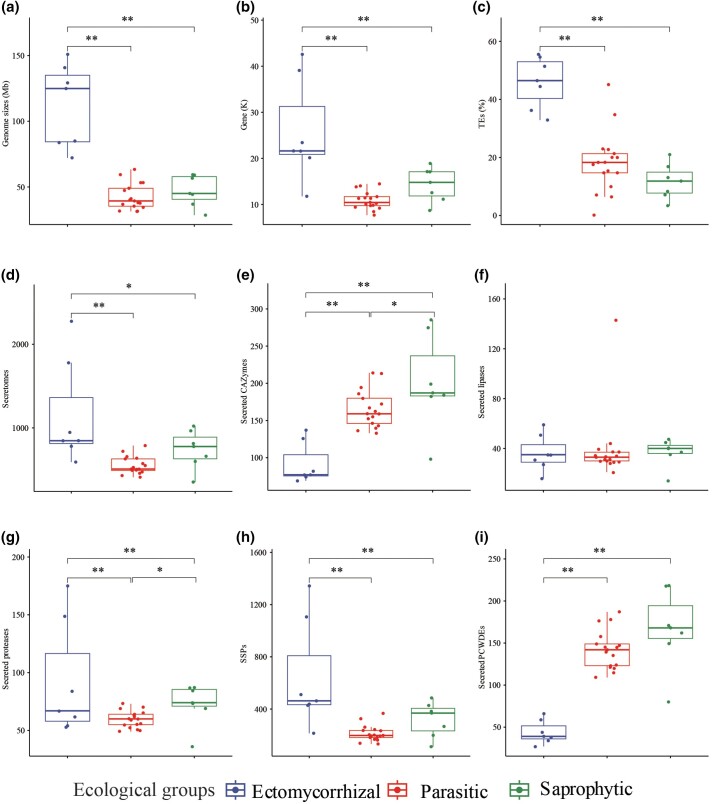
Genome features in three ecological groups—ectomycorrhizal, saprophytic, and parasitic species, respectively. (*a*) Genome size (Mb). (*b*) Numbers of gene models (thousands). (*c*) Percentage of TEs in entire genomes. (*d*) Numbers of secretome proteins. (*e*) Numbers of secreted carbohydrate-active enzymes (CAZymes). (*f*) Numbers of secreted lipases. (*g*) Numbers of secreted proteases. (*h*) Numbers of SSPs (with less than 300 amino acids). (*i*) Numbers of secreted PCWDEs. * and ** indicate significant difference between ecological groups with *P* < 0.05 and < 0.01, respectively.

The size of the genome assembly of the 31 genomes in Hymenochaetales ranged from 28.6 Mb in *Phellinidium pouzarii* DSM 108285 to 150.86 Mb in *C. weii* Dai 23719 with GC contents ranging from of 40.83% in *Fomitiporia mediterranea* MF3-22 to 52.43% in *Fuscoporia viticola* PhevitSig-SM15, and with number of predicted gene coding models ranging from 7,690 in *Inonotus obliquus* CT5 to 42,579 in *C. weii* Dai 23719 (table 1 and fig. 2*b*). From 84.1% in *Coltricia abieticola* Dai 22737 to 95.5% in *Trichaptum fuscoviolaceum* of BUSCO were found in 31 genomes of the Hymenochaetales (table 1 and fig. 2*b*), suggesting that genomes captured most of the protein-coding gene space. In addition, the genomes of the ectomycorrhizal species are significantly larger than the parasitic and saprophytic species in assembly size and number of protein-coding gene models, while no significant differences were observed between parasitic and saprophytic species (fig. 3*a* and *b*; *P* < 0.01).

### Phylogenomic Relationships

A maximum likelihood (ML) phylogenomic analysis of the 32 genomes was carried out under the tentatively best substitution model PROTGAMMAIJTTF (supplementary Files S2 and S3, Supplementary Material online). The generated Maximum Clade Credibility (MCC) tree suggests that the ectomycorrhizal genus *Coltricia* forms a clade located at the base of the Hymenochaetaceae (fig. 2*a*), and the divergence time estimation suggested that *Coltricia* occurred at a mean stem age of 105.9 Mya, most extant species of Hymenochaetales diversified at no more than 20 Mya. However, parasitic and saprophytic species of Hymenochaetaceae did not form independent clades. It showed that two parasitic/saprophytic species, *Coniferiporia sulphurascens* and *F. viticola*, clustered with saprophytic species in Hymenochaetaceae, which is in agreement with previous phylogenetic studies (Dai 2010; Wu et al. 2022b).

### Transposable Elements

In the 31 Hymenochaetales genomes, the TE contents varied from 0.14% in *F. viticola* PhevitSig-SM15 to 55.51% in *C. abieticola* Dai 22736. The long terminal repeats, such as *Gypsy* and *Copia*, were the most abundant in *Coltricia* (fig. 2*b* and supplementary File S1, Supplementary Material online).

Most notably, the ectomycorrhizal genus *Coltricia* has the largest genomes with significantly higher TE contents compared with those of parasitic and saprophytic genera in the Hymenochaetales (fig. 3*c*; *P* < 0.01), while there were no significant differences (*P* > 0.05) in the average TE contents between parasitic and saprophytic fungi (fig. 3*c*). TE contents ranged from 32.89% in *C. perennis* Dai 23027 to 55.51% in *C. abieticola* Dai 22736 in the ectomycorrhizal species, but from 0.14% in *F. viticola* PhevitSig-SM15 to 45.10% in *F. mediterranea* in the parasitic species, and from 3.4% in *Xylodon ovisporus* KUC8140 to 20.99% in *T. fuscoviolaceum* in the saprophytic species, respectively (fig. 2*b*).

### The Predicted Secreted Proteins

**Fig. 4:**
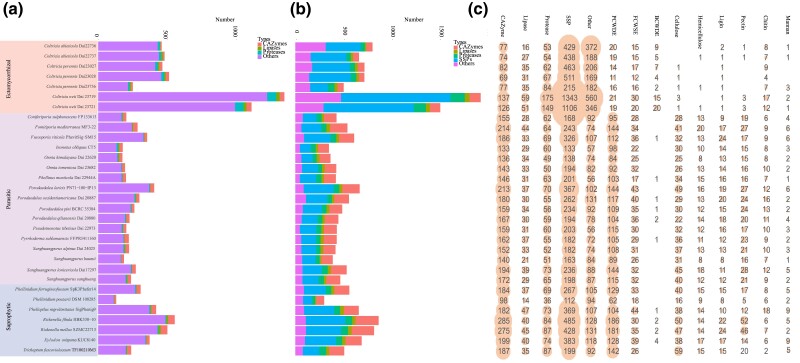
Secreted proteins profiles of 31 Hymenochaetales. (*a*) Distribution of SSPs in CAZymes, lipases, proteases, and others. (*b*) Distribution of secretome in CAZymes, lipases, proteases, SSPs, and others. (*c*) The number of genes for secreted CAZymes, secreted lipases, secreted proteases, SSPs, others secreted, secreted plant cell wall degrading enzymes, secreted fungal cell wall degrading enzymes, secreted BCWDE, degrading enzymes of cellulose, hemicellulose, lignin, pectin, chitin, and mannan. Bars represent the gene copy numbers.

Ectomycorrhizal species in the Hymenochaetales have also a significantly larger secretome compared with those of parasitic and saprophytic species (fig. 3*d*; *P* < 0.01). small secreted proteins (SSPs) had the most abundant profile in secreted proteins, followed by CAZymes, proteases, and lipases, accounting for 31.6–62.2%, 6.0–50.0%, 5.6–14.6%, and 1.4–30.1%, respectively. Although SSPs are the main profile of secreted proteins, a small amount of SSPs is annotated as CAZymes, lipases, and proteases (fig. 4). Notably, ectomycorrhizal species have significantly higher secreted proteins, SSPs, and secreted proteases compared with those of parasitic and saprophytic fungi (fig. 3*d*, *g,* and *h*; *P* < 0.01). while smaller secreted CAZymes of ectomycorrhizal species have identified (fig. 3*e*; *P* < 0.01). And ectomycorrhizal species have no difference in secreted lipases (fig. 3*f*; *P* > 0.05) compared with those of parasitic and saprophytic fungi. In addition, saprophytic species have significantly higher secreted CAZymes and secreted proteases (fig. 3*e* and *g*; *P* < 0.05), but less secreted proteins, secreted lipases, and SSPs (fig. 3*d*, *f*, and *h*).

Secreted CAZymes play an important role in wood degradation, and our analyses show that the average number of secreted CAZymes in ectomycorrhizal species is significantly less than that in parasitic and saprophytic species (91.7 ± 25.6 vs. 165.4 ± 24.2 and 201.4 ± 58.5, respectively; figs. 3*e* and 4*c*). Among these secreted CAZymes (supplementary fig. S1, Supplementary Material online), ectomycorrhizal species are significantly different from parasitic and saprophytic species regarding the gene number of auxiliary activities (AAs), carbohydrate-binding modules (CBMs), glycoside hydrolases (GHs), and glycosyl transferases, while less difference is found in carbohydrate esterases (CEs).

### PCWDEs Loss in Ectomycorrhizal *Coltricia*

**Fig. 5:**
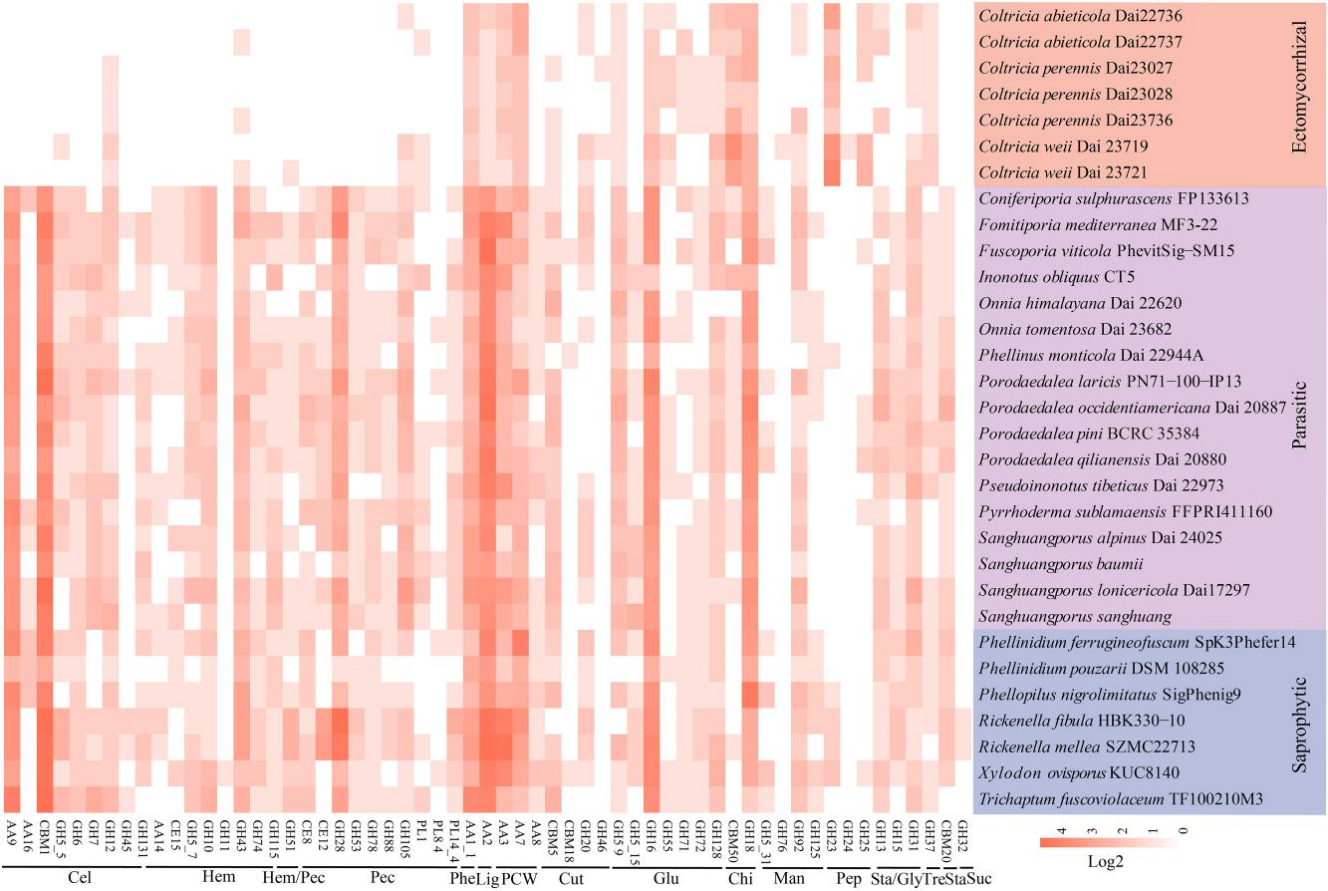
Distribution of classical secreted CAZymes in 31 Hymenochaetales genomes in three ecological groups. The CAZyme genes identified correspond to eight AAs, five CBMs, three CEs, 40 glycoside hydrolase (GHs), and three PLs families. The potential substrates are cellulose, hemicellulose, lignin, and pectin for plant cell walls; chitin, glucan, and mannan for fungal cell walls, and peptidoglycan for bacterial cell walls. Cel, cellulose; Hem, hemicellulose; Pec, pectin; Phe, phenols; Lig, lignin; PCW, partial plant cell wall degradation; Cut, cutin; Glu, glucan; Chi, chitin; Man, mannan; Pep, peptidoglycan; Sta, starch; Gly, glycogen; Tre, trehalose; Suc, sucrose.

In this study, a total of 59 secreted CAZymes families, including eight AAs families, five CBMs families, three CEs families, 40 GHs families, and three polysaccharide lyase (PLs) families, is annotated as secreted plant, fungal, or bacterial cell wall degrading enzymes (BCWDE) (fig. 5 and supplementary File S4, Supplementary Material online). The gene copy number for secreted PCWDEs of ectomycorrhizal species showed striking differences to the parasitic and saprophytic species (figs. 2*i* and 5; *P* < 0.01). The number of secreted PCWDEs in ectomycorrhizal species ranged from 11 in *C. perennis* Dai 23028 to 21 in *C. weii* Dai 23719, but 84–144 and 62–186 in parasitic and saprophytic species, respectively (fig. 4*c*).

We classified the gene families involved in secreted cell wall degradation enzymes into 17 main categories as described in previous studies (Sipos et al. 2017; Wu et al. 2022c). Compared with those of parasitic and saprophytic species, ectomycorrhizal species have almost lost the capacity to degrade cellulose, hemicellulose, and pectin (fig. 4*c*, fig. 5 and supplementary File S4, Supplementary Material online). For example, *Coltricia* species have completely lost glycoside hydrolase (GH6 and GH7 families), and cellulose-binding motif (CBM1 family, core cellulose-acting CAZymes attached to PCWDEs to mediate the targeting of enzymes to cellulose; Martínez et al. 2018; Wu et al. 2022c), which, however, are over-represented in parasitic and saprophytic species. From 11 to 21, secreted PCWDEs were predicted in the ectomycorrhizal *Coltricia* species, indicating a limited capacity to degrade plant cell walls. Concerning lignin degradation, the ectomycorrhizal species had one to two genes of AA2 family (class II lignin-modifying PODs) annotated, while parasitic and saprophytic species had 8 to 24 genes for the AA2 family. However, the ectomycorrhizal species have more genes to degrade chitin (CBM50 and GH18 families) and peptidoglycan (GH23 family), indicating that they have a stronger ability to degrade fungal and bacterial cell walls.

On the other hand, a total of 68 CAZyme families (including secreted and nonsecreted) are classified as cell wall degrading enzymes, and 164–445, 236–353, and 204–368 genes were annotated in ectomycorrhizal, parasitic, and saprophytic species, respectively (supplementary figs. S2 and S3, Supplementary Material online, supplementary Files S5, Supplementary Material online). A total of 303 CAZyme families or subfamilies is annotated in the 31 Hymenochaetales genomes (supplementary File S6, Supplementary Material online).

### The Relationship Between Hymenochaetales Species and Host Trees

We analyzed the secreted CAZyme families of cellulose and lignin in Hymenochaetales species, which grown on gymnosperms or angiosperms, including AA9, AA16, CBM1, GH5_5, GH6, GH7, GH12, GH 45, GH131, and AA2 (supplementary fig. S4, Supplementary Material online). AA2 family plays a major role in the degradation of lignin content in plant cell walls. Although relatively more genes of AA2 families in species growing on gymnosperms were detected than in those growing on angiosperms, this difference was not significant (supplementary fig. S4*a*, Supplementary Material online; *P* > 0.05).

**Fig. 6:**
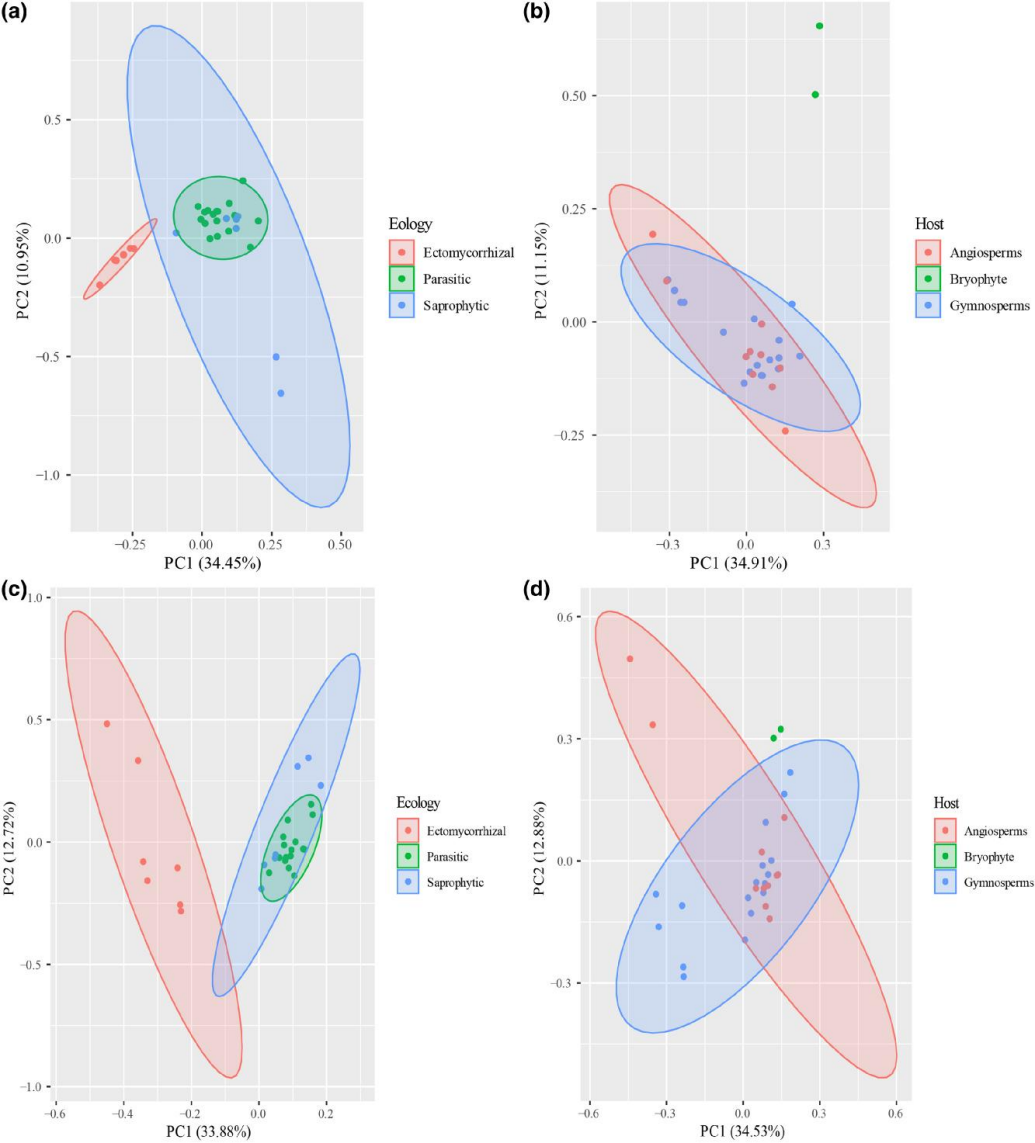
PCA of ecological groups and CAZyme domains (*a*), host plants and secreted CAZyme domains (*b*), ecological groups and total CAZyme domains (*c*), and host plants and total CAZyme domains (*d*) in 31 Hymenochaetales genomes.

The results showed that no significant difference is observed in the numbers of genes of nine gene families involved in cellulose degradation (supplementary fig. S4*b*–*l*, Supplementary Material online; *P* > 0.05). Principal component analyses (PCA; fig. 6) of CAZymes (secreted and nonsecreted) suggest that ectomycorrhizal species are significantly different from parasitic and saprophytic species, which is probably related to the ecology of the fungi. The type of host plant (whether angiosperms or gymnosperms) has a less influence, except for *Rickenella* species associated with bryophyte (fig. 6). In addition, the PCA analyses of host plants with CAZymes (secreted and nonsecreted) were preformed, the results showed the limited patterns observed that no significant differences to parasitic-only and saprotrophic-only species grown on angiosperms and gymnosperms (supplementary fig. S5, Supplementary Material online).

### *Rickenella fibula* and *Rickenella mellea* as Special Saprophytic Fungi

*Rickenella fibula* and *R. mellea* are very similar to saprotrophic species in the Hymenochaetales in terms of genome size, gene models, TE contents, secreted proteins, and cell wall degrading enzymes (table 1, supplementary Files S3 and S4 and S5, Supplementary Material online) but are significantly different from species of the ectomycorrhizal genus *Coltricia*. The gene copy numbers of secreted PWCDEs in *R. fibula* and *R. mellea* are 186 and 181, respectively (fig. 4), especially mainly concentrated in the AA1_1, AA2, AA7, AA9, CBM1, CE12, GH5_5, GH15, GH16, GH18, GH28, GH43, and PL14_4 families, suggesting that both *R. fibula* and *R. mellea* have a powerful capacity for degrading plant cell walls. And *R. fibula* and *R. mellea* have a single gene copy of secreted GH32 family, which indicates they have the ability to degrade sucrose. Korotkin et al. suggested *R. fibula* appear to have multiple trophic modes and most likely maintaining a commensal endophytic relationship with its moss host. (Korotkin 2017; Korotkin et al. 2018). Considering the ancestors were saprotrophic, and *R. fibula* and *R. mellea* have a large number of PCWDEs, inferring that they are as special saprophytic fungi.

## Discussion

In the fungal kingdom, ectomycorrhizal fungi have evolved independently in 78–82 fungal lineages that comprise 251–256 genera (Tedersoo and Smith 2013; Martin et al. 2016). Phylogenetic analysis of 8,400 species within Agaricomycetes suggested 36 ectomycorrhizal origins (Sánchez-García et al. 2020), among which many EcM are located in the orders Agaricales, Boletales, Cantharellales, and Russulales, while only a few are found in the Hymenochaetales, that is, *Coltricia* species (Sánchez-García et al. 2020; Hackel et al. 2022; Wu et al. 2022b, 2022c). Comparative genomics indicated that the ancestors of EcM were ecologically diverse, including brown rot, white rot, and soil saprotrophs (Kohler et al. 2015; Miyauchi et al. 2020; Lebreton et al. 2021; Wu et al. 2022c). Here, phylogenomic and molecular clock analyses based on 31 Hymenochaetales genomes showed that ectomycorrhizal *Coltricia* is located at the base of the Hymenochaetaceae and divergence time of *Coltricia* later than the saprotrophic species. Interestingly, brown rot and their EcM decedents, such as ectomycorrhizal Boletales and Atheliales/Amylocorticiales, lack the secreted AA2 family (Wu et al. 2022c), while a single to two gene copies of AA2 family is predicted in *Coltricia*, inferring that *Coltricia* may have originated from saprotrophic ancestor with white rot.

A distinguishing character of EcM related to white rot and brown rot fungi is the loss of several families of PCWDEs, especially those acting on cellulose and lignin (such as AA2, GH6, and GH7 families). Unique array of PCWDEs for the ectomycorrhizal fungi was found, such as GH5 endoglucanases with a CBM1 cellulose-binding motif, pectinases (GH28 family), oxidoreductases, and laccases (AA1 and AA9 families; Kohler et al. 2015; Martin et al. 2016; Lebreton et al. 2021; Sun et al. 2022; Wu et al. 2022c). In this study, we found that the gene copy number for secreted PCWDEs in *Coltricia* species is also dramatically reduced, as reported for ectomycorrhizal orders Boletales, Russulales, Thelephorales, and family Amanitaceae (Hess et al. 2018; Miyauchi et al. 2020; Lofgren et al. 2021; Looney et al. 2021; Wu et al. 2022c). *Coltricia* species completely lost the CBM1, GH6, and GH7 families, which is a significant difference to those of the parasitic and saprophytic relatives.

In addition, the genomes of saprotrophic Hymenochaetales encode a larger PCWDEs repertoire (62–186 genes) than Boletales (100–148 genes), while slightly less than Agaricales (122–338 genes), Polyporales (89–212 genes), and Russulales (160–253 genes; Floudas et al. 2020; Ruiz-Dueñas et al. 2020; Lebreton et al. 2021). Our analyses of *Coltricia* PCWDEs support the view that the transition to symbiosis entailed the loss of PCWDEs acting on cellulose, hemicellulose, pectin, and lignin.

Additional genome traits, such as the content in transposable elements (TEs), SSPs have also been investigated in EcM (Hess et al. 2018; Miyauchi et al. 2020; Lofgren et al. 2021; Looney et al. 2021; Wu et al. 2022c). Our results show that TE and SSP content in the genome of ectomycorrhizal *Coltricia* species are also enriched. In addition, rapidly dynamic genomes with higher gene models are observed in *Coltricia*, similar to the genus *Suillus* (Lofgren et al. 2021). However, more genomes within the Hymenochaetales should be sequenced and analyzed to assist in studying evolutionary diversification because of the richness of species in the order.

The ecological groups (saprophytic and parasitic) and the corresponding host trees (gymnosperms and angiosperms) of Hymenochaetales species have highly similar genome features, including PCWDEs, secreted CAZymes and TEs, and are not divided into two independent groups based on their genome comparison. Despite differences in cellulose and lignin profiles between gymnosperms and angiosperms (Cornwell et al. 2009; Thakur and Thakur 2014), we revealed no significant differences in secreted CAZymes of Hymenochaetales species growing on the different host trees, inferred that the formation of secreted CAZymes of Hymenochaetales is earlier than the differentiation of the host trees and there are other recognition mechanisms for fungi allowing growth on either gymnosperms or angiosperms. Molecular dating analyses have suggested that the divergence times of Hymenochaetales and Basidiomycota are 167–259 Mya and more than 400 Mya, respectively (He et al 2019; Varga et al. 2019; Zhao et al. 2022b). In contrast, the host trees, viz., gymnosperms and angiosperms, can be traced back to the Carboniferous period 300–350 Mya (Won and Renner 2006; Clarke et al. 2011; Magallón et al. 2013; Wang and Ran 2014), which later than the origin of Basidiomycota. In addition, some studies have suggested that many factors, such as Pi transporters, chitotetraose, receptor-like kinase, lipochitooligosaccharides, and SSPs, could be involved in the formation of an ectomycorrhizal life mode (Plett et al. 2011; Becquer et al. 2018; Cope et al. 2019; Pellegrin et al. 2019; Zhang et al. 2021), and these may be involved in the recognition mechanism to grow on gymnosperms or angiosperms.

To conclude, the 15 new genomes of Hymenochaetaceae were sequenced in this study, including important ectomycorrhizal *Coltricia*, provided valuable data for phylogenomic and genomic analyses to explain evolutionary innovations in the order Hymenochaetales.

## Materials and Methods

### Genome Collection

In this study, a total of 32 genomes was collected, including 17 genomes downloaded from NCBI (https://www.ncbi.nlm.nih.gov/genome; accessed on November 14, 2022) and JGI MycoCosm database (https://mycocosm.jgi.doe.gov; accessed on November 14, 2022), and 15 sequenced by the authors (table 1 and fig. 1). The 15 newly sequenced genomes represent 11 species of six genera, including *Coltricia*, *Onnia*, *Phellinus*, *Porodaedalea*, *Pseudoinonotus*, and *Sanghuangporus*, within Hymenochaetaceae; all the sequences were deposited to the NCBI database (BioProject ID: PRJNA988840, and BioSample accessions: SAMN36028741–SAMN36028755).

### DNA Extraction, Genome Sequencing, and Assembly

Genomic DNA of fresh basidiocarps was extracted using a kit O-GPLF-400 (GeneOnBio Co., Changchun, China), following the manufacturer's protocol. Total DNAs were detected using DNA/Protein Analyzer and 1% agarose gel electrophoresis. High-quality DNA samples were sequenced at Tsingke Biotechnology Co., Ltd. (Beijing, China) using the Illumina NovaSeq 6000 platform with a 350 bp library using pair-end sequencing. And the *k*-mer analyses were carried out with Jellyfish v2.3.0 (Marçais and Kingsford 2011) and GenomeScope v1.0 (Vurture et al. 2017) with default paramenters (supplementary File S7, Supplementary Material online). Low-quality reads (contaminated and duplication reads, less than 50 bp long, or unknown bases exceeding 50%) were removed and then de novo assembled using MaSuRCA v3.4.3b (Zimin et al. 2013) with default paramenters. Finally, the quality of the assembled genomes was assessed by Quast v5.0.2 (Gurevich et al. 2013) and BUSCO v5.2.2 with the Agaricomycetes gene set downloaded from https://busco-data.ezlab.org/v5/data/lineages/agaricomycetes_odb10.2020-08-05.tar.gz, accessed on June 16, 2022 (Simão et al. 2015).

### Gene Prediction and Functional Annotation

Gene models of the 15 newly sequenced genomes were de novo predicted by Augustus v3.3.3 (Stanke and Waack 2003) with default paramenters due to a lack of reference genomes. Protein-coding gene models were functionally analyzed using Carbohydrate-Active Enzymes (CAZymes; Cantarel et al. 2009), Cluster of Orthologous Groups of proteins (Galperin et al. 2015), evolutionary gene genealogy Nonsupervised Orthologous Groups (eggNOG; Huerta-Cepas et al. 2019), gene ontology (Ashburner et al. 2000), NR (https://www.ncbi.nlm.nih.gov/protein, accessed on October 15, 2022), Kyoto Encyclopedia of Genes and Genomes (KEGG; Kanehisa et al. 2016), and Pfam (El-Gebali et al. 2019) databases. And all the functional annotations use the Diamond v2.0.2 (Buchfink et al. 2015) with a cutoff values of E-value no more than 1 × 10^−5^.

Secreted proteins of 31 Hymenochaetales genomes were predicted as described by Pellegrin et al. (2015). In brief, first, signal peptides are detected with SignalP v5.0 (Almagro et al. 2019). Then, those with a transmembrane helix were removed using DeepTMHMM v1.0.18 (Hallgren et al. 2022), and those with a subcellular localization that permanently resides in the endoplasmic reticulum and KDEL amino acids (Lys-Asp-Glu-Leu motifs in the C-terminal region), were filtered out using TargetP v2.0 (Emanuelsson et al. 2000). Proteins less than 300 amino acids long were considered as SSPs. The secreted carbohydrate-active enzymes, lipases, and proteases were annotated with CAZymes (Cantarel et al. 2009), Lipase Engineering Database (Fischer and Pleiss 2003), and MEROPS database (Rawlings et al. 2010). TEs were identified using the Extensive de novo TE Annotator pipeline v1.9.5 (Ou et al. 2019) with the paramenters (−overwrite 1 –sensitive 1 –anno 1 –evaluate 1 –force 1 –threads 10). CAZyme domains were classified as degraded of cellulose, hemicellulose, lignin, pectin, chitin, and mannan by previous studies (Sipos et al. 2017; Wu et al. 2022c).

### Phylogenomic and Divergence Time Analyses

A total of 32 genomes, including 31 Hymenochaetales and one genome of *Agaricus bisporus* as outgroup, were used to reconstruct phylogenomic relationships based on single-copy orthologous genes (table 1). These genes were found using OrthoFinder v2.5.4 (Emms and Kelly 2019) and aligned using MAFFT v7 (Katoh and Standley 2013); those alignments that covered less than 50 amino acids or poorly aligned were excluded. A ML phylogenomic tree was reconstructed by RAxML v8.1.12 (Stamatakis 2014) with 100 bootstrap replications. The best substitution model was estimated using ModelTest-NG v0.1.7 (Darriba et al. 2020). The divergence time was estimated using r8s v1.71 (Sanderson 2003) based on the single-copy orthologous genes. The calibration of Hymenochaetales was 167 Mya with the 130 Mya of min-age and 180 Mya of max-age (Varga et al. 2019), and the penalized likelihood method was selected. Finally, the ML and MCC trees were viewed with FigTree v1.4.4 (http://tree.bio.ed.ac.uk/software/figtree; accessed on May 1, 2020).

## Supplementary Material

evad136_Supplementary_Data

## Data Availability

The heatmaps of CAZymes domains (secreted or total) were preformed by the ImageGP (https://www.bic.ac.cn/ImageGP/; Chen et al. 2022). Drawing and principal component analysis using ggfortify and ggplot2 packages in R (Ihaka and Gentleman 1996; Wickham 2011; Tang et al. 2016). All newly generated sequences have been deposited in the NCBI database under the BioProject ID: PRJNA988840 and BioSample accessions: SAMN36028741–SAMN36028755.

## References

[evad136-B1] Almagro AJJ , et al 2019. Signalp 5.0 improves signal peptide predictions using deep neural networks. Nat Biotechnol. 37:420–423. 30778233 10.1038/s41587-019-0036-z

[evad136-B2] Ashburner M , et al 2000. Gene ontology: tool for the unification of biology. Nat Genet. 25:25–29. 10802651 10.1038/75556 PMC3037419

[evad136-B3] Becquer A , et al 2018. The *Hebeloma cylindrosporum* HcPT2 Pi transporter plays a key role in ectomycorrhizal symbiosis. New Phytol. 220:1185–1199. 29944179 10.1111/nph.15281

[evad136-B4] Bian LS , Zhou M, Yu J. 2022. Three new *Coltricia* (Hymenochaetaceae, Basidiomycota) species from China based on morphological characters and molecular evidence. Mycol Progress. 21:45.

[evad136-B5] Buchfink B , Xie C, Huson D. 2015. Fast and sensitive protein alignment using DIAMOND. Nat Methods. 12:59–60. 25402007 10.1038/nmeth.3176

[evad136-B6] Cantarel BL , et al 2009. The Carbohydrate-Active EnZymes database (CAZy): an expert resource for glycogenomics. Nucleic Acids Res. 37:D233–D238. 18838391 10.1093/nar/gkn663 PMC2686590

[evad136-B7] Chen T , Liu YX, Huang LQ. 2022. ImageGP: an easy-to-use data visualization web server for scientific researchers. iMeta 1:e5. 10.1002/imt2.5 PMC10989750 38867732

[evad136-B8] Chung CL , et al 2017. Comparative and population genomic landscape of *Phellinus noxius*: a hypervariable fungus causing root rot in trees. Mol Ecol. 26:6301–6316. 28926153 10.1111/mec.14359

[evad136-B9] Clarke JT , Warnock RCM, Donoghue PCJ. 2011. Establishing a time-scale for plant evolution. New Phytol. 192:266–301. 21729086 10.1111/j.1469-8137.2011.03794.x

[evad136-B10] Cope KR , et al 2019. The ectomycorrhizal fungus *Laccaria bicolor* produces lipochitooligosaccharides and uses the common symbiosis pathway to colonize *Populus* roots. Plant Cell. 31:2386–2410. 31416823 10.1105/tpc.18.00676 PMC6790088

[evad136-B11] Cornwell WK , et al 2009. Plant traits and wood fates across the globe: rotted, burned, or consumed? Global Change Biol. 15:2431–2449.

[evad136-B12] Dai YC . 2010. Hymenochaetaceae (Basidiomycota) in China. Fungal Divers. 45:131–343.

[evad136-B13] Danielson RM . 1984. Ectomycorrhizal associations in jack pine stands in northeastern Albcrta. Can J Bot. 62:932–939.

[evad136-B14] Darriba D , et al 2020. ModelTest-NG: a new and scalable tool for the selection of DNA and protein evolutionary models. Mol Biol Evol. 37:291–294. 31432070 10.1093/molbev/msz189 PMC6984357

[evad136-B15] Duan Y , et al 2022. Genome sequencing of *Inonotus obliquus* reveals insights into candidate genes involved in secondary metabolite biosynthesis. BMC Genomics. 23:314. 35443619 10.1186/s12864-022-08511-x PMC9020118

[evad136-B16] El-Gebali S , et al 2019. The Pfam protein families database in 2019. Nucleic Acids Res. 47:D427–D432. 30357350 10.1093/nar/gky995 PMC6324024

[evad136-B17] Emanuelsson O , Nielsen H, Brunak S, Von Heijne G. 2000. Predicting subcellular localization of proteins based on their N-terminal amino acid sequence. J Mol Biol. 300:1005–1016. 10891285 10.1006/jmbi.2000.3903

[evad136-B18] Emms DM , Kelly S. 2019. Orthofinder: phylogenetic orthology inference for comparative genomics. Genome Biol. 20:238. 31727128 10.1186/s13059-019-1832-y PMC6857279

[evad136-B19] Fernández-López J , Martín MP, Dueñas M, Telleria MT. 2018. Multilocus phylogeny reveals taxonomic misidentification of the *Schizopora paradoxa* (KUC8140) representative genome. MycoKeys 38:121–127. 10.3897/mycokeys.38.28497 PMC6160840 30275743

[evad136-B20] Fischer M , Pleiss J. 2003. The Lipase Engineering Database: a navigation and analysis tool for protein families. Nucleic Acids Res. 31:319–321. 12520012 10.1093/nar/gkg015 PMC165462

[evad136-B21] Floudas D , et al 2012. The Paleozoic origin of enzymatic lignin decomposition reconstructed from 31 fungal genomes. Science 336:1715–1719. 22745431 10.1126/science.1221748

[evad136-B22] Floudas D , et al 2020. Uncovering the hidden diversity of litter-decomposition mechanisms in mushroom-forming fungi. ISME J. 14:2046–2059. 32382073 10.1038/s41396-020-0667-6 PMC7368018

[evad136-B23] Galperin MY , Makarova KS, Wolf YI, Koonin EV. 2015. Expanded microbial genome coverage and improved protein family annotation in the COG database. Nucleic Acids Res. 43:D261–D269. 25428365 10.1093/nar/gku1223 PMC4383993

[evad136-B24] Gurevich A , Saveliev V, Vyahhi N, Tesler G. 2013. QUAST: quality assessment tool for genome assemblies. Bioinformatics 29:1072–1075. 23422339 10.1093/bioinformatics/btt086 PMC3624806

[evad136-B25] Hackel J , et al 2022. Biogeographic history of a large clade of ectomycorrhizal fungi, the Russulaceae, in the Neotropics and adjacent regions. New Phytol. 236:698–713. 35811430 10.1111/nph.18365 PMC9795906

[evad136-B26] Hallgren J , et al 2022. DeepTMHMM predicts alpha and beta transmembrane proteins using deep neural networks. bioRxiv. 10.1101/2022.04.08.487609, preprint: not peer reviewed.

[evad136-B27] Hawksworth DL , Lücking R. 2017. Fungal diversity revisited: 2.2 to 3.8 million species. Microbiol Spectr. 5:1–17. 10.1128/microbiolspec.funk-0052-2016 PMC11687528 28752818

[evad136-B28] He MQ , et al 2019. Notes, outline and divergence times of Basidiomycota. Fungal Divers. 99:105–367.

[evad136-B29] Heimann M , Reichstein M. 2008. Terrestrial ecosystem carbon dynamics and climate feedbacks. Nature 451:289–292. 18202646 10.1038/nature06591

[evad136-B30] Hess J , et al 2018. Rapid divergence of genome architectures following the origin of an ectomycorrhizal symbiosis in the genus *Amanita*. Mol Biol Evol. 35:2786–2804. 30239843 10.1093/molbev/msy179 PMC6231487

[evad136-B31] Huerta-Cepas J , et al 2019. eggNOG 5.0: a hierarchical, functionally and phylogenetically annotated orthology resource based on 5090 organisms and 2502 viruses. Nucleic Acids Res. 47:D309–D314. 30418610 10.1093/nar/gky1085 PMC6324079

[evad136-B32] Caballero JRI , et al 2020. Genome comparison and transcriptome analysis of the invasive brown root rot pathogen, *Phellinus noxius*, from different geographic regions reveals potential enzymes associated with degradation of different wood substrates. Fungal Biol. 124:144–154. 32008755 10.1016/j.funbio.2019.12.007

[evad136-B33] Ihaka R , Gentleman R. 1996. R: a language for data analysis and graphics. J Comput Graph Stat. 5:299–314.

[evad136-B34] Jiang JH , Wu SH, Zhou LW. 2021. The first whole genome sequencing of *Sanghuangporus sanghuang* provides insights into its medicinal application and evolution. J Fungi (Basel). 7:787. 34682209 10.3390/jof7100787 PMC8537844

[evad136-B35] Kanehisa M , Sato Y, Kawashima M, Furumichi M, Tanabe M. 2016. KEGG as a reference resource for gene and protein annotation. Nucleic Acids Res. 44:D457–D462. 26476454 10.1093/nar/gkv1070 PMC4702792

[evad136-B36] Katoh K , Standley DM. 2013. MAFFT: multiple sequence alignment software version 7: improvements in performance and usability. Mol Biol Evol. 30:772–780. 23329690 10.1093/molbev/mst010 PMC3603318

[evad136-B37] Kohler A , et al 2015. Convergent losses of decay mechanisms and rapid turnover of symbiosis genes in mycorrhizal mutualists. Nat Genet. 47:410–415. 25706625 10.1038/ng.3223

[evad136-B38] Korotkin HB . 2017. Stable isotopes, phylogenetics, and experimental data indicate a unique nutritional mode for *Rickenella fibula*, a bryophyte-associate in the Hymenochaetales. Knoxville: University of Tennessee.

[evad136-B39] Korotkin HB , et al 2018. Stable isotope analyses reveal previously unknown trophic mode diversity in the Hymenochaetales. Am J Bot. 105:1869–1887. 30368779 10.1002/ajb2.1183

[evad136-B40] Krah FS , et al 2018. Evolutionary dynamics of host specialization in wood-decay fungi. BMC Evol Biol. 18:1–13. 30075699 10.1186/s12862-018-1229-7 PMC6091043

[evad136-B41] Krizsán K , et al 2019. Transcriptomic atlas of mushroom development reveals conserved genes behind complex multicellularity in fungi. Proc Natl Acad Sci U S A. 116:7409–7418. 30902897 10.1073/pnas.1817822116 PMC6462078

[evad136-B42] Lebreton A , et al 2021. Evolution of the mode of nutrition in symbiotic and saprotrophic fungi in forest ecosystems. Ann Rev Ecol Evol Syst. 52:385–404.

[evad136-B43] Lee HH , et al 2019. Evidence of extensive intraspecific noncoding reshuffling in a 169-kb mitochondrial genome of a Basidiomycetous fungus. Genome Bio Evol. 11:2774–2788. 31418013 10.1093/gbe/evz181 PMC6786477

[evad136-B44] Lofgren LA , et al 2021. Comparative genomics reveals dynamic genome evolution in host specialist ectomycorrhizal fungi. New Phytol. 230:774–792. 33355923 10.1111/nph.17160 PMC7969408

[evad136-B45] Looney B , Miyauchi S, Morin E, Drula E, Courty PE, Kohler A, Kuo A, LaButti K, Pangilinan J, Lipzen A. 2021. Evolutionary priming and transition to the ectomycorrhizal habit in an iconic lineage of mushroom-forming fungi: is preadaptation a requirement?. bioRxiv. 2021.2002.2023.432530. 10.1101/2021.02.23.432530, preprint: not peer reviewed.

[evad136-B46] Magallón S , Hilu KW, Quandt D. 2013. Land plant evolutionary timeline: gene effects are secondary to fossil constraints in relaxed clock estimation of age and substitution rates. Am J Bot. 100:556–573. 23445823 10.3732/ajb.1200416

[evad136-B47] Marçais G , Kingsford C. 2011. A fast, lock-free approach for efficient parallel counting of occurrences of *k*-mer. Bioinformatics 27:764–770. 21217122 10.1093/bioinformatics/btr011 PMC3051319

[evad136-B48] Martin F , Kohler A, Murat C, Veneault-Fourrey C, Hibbett DS. 2016. Unearthing the roots of ectomycorrhizal symbioses. Nat Rev Microbiol. 14:760–773. 27795567 10.1038/nrmicro.2016.149

[evad136-B49] Martínez A , Camarero S, Ruiz-Dueñas F, Martínez M. 2018. Biological lignin degradation. In: Beckham GT, editor. Lignin valorization: emerging approaches. London: Royal Society of Chemistry. p. 199–225.

[evad136-B50] Min B , et al 2015. Genome sequence of a white rot fungus *Schizopora paradoxa* KUC8140 for wood decay and mycoremediation. J Biotechnol. 211:42–43. 26188242 10.1016/j.jbiotec.2015.06.426

[evad136-B51] Miyauchi S , et al 2020. Large-scale genome sequencing of mycorrhizal fungi provides insights into the early evolution of symbiotic traits. Nat Commun. 11:1–17. 33046698 10.1038/s41467-020-18795-w PMC7550596

[evad136-B52] Morin E , et al 2012. Genome sequence of the button mushroom *Agaricus bisporus* reveals mechanisms governing adaptation to a humic-rich ecological niche. Proc Natl Acad Sci U S A. 109:17501–17506. 23045686 10.1073/pnas.1206847109 PMC3491501

[evad136-B53] Naranjo-Ortiz MA , Gabaldón T. 2019. Fungal evolution: major ecological adaptations and evolutionary transitions. Biol Rev. 94:1443–1476. 31021528 10.1111/brv.12510 PMC6850671

[evad136-B54] Ou S , et al 2019. Benchmarking transposable element annotation methods for creation of a streamlined, comprehensive pipeline. Genome Biol. 20:1–18. 31843001 10.1186/s13059-019-1905-y PMC6913007

[evad136-B55] Pellegrin C , et al 2019. *Laccaria bicolor* MiSSP8 is a small-secreted protein decisive for the establishment of the ectomycorrhizal symbiosis. Environ Microbiol. 21:3765–3779. 31260142 10.1111/1462-2920.14727

[evad136-B56] Pellegrin C , Morin E, Martin FM, Veneault-Fourrey C. 2015. Comparative analysis of secretomes from ectomycorrhizal fungi with an emphasis on small-secreted proteins. Front Microbiol. 6:1278. 26635749 10.3389/fmicb.2015.01278 PMC4649063

[evad136-B57] Plett JM , et al 2011. A secreted effector protein of *Laccaria bicolor* is required for symbiosis development. Curr Biol. 21:1197–1203. 21757352 10.1016/j.cub.2011.05.033

[evad136-B58] Rawlings ND , Barrett AJ, Bateman A. 2010. MEROPS: the peptidase database. Nucleic Acids Res. 38(Suppl 1):D227–D233. 19892822 10.1093/nar/gkp971 PMC2808883

[evad136-B59] Ruiz-Dueñas FJ , et al 2020. Genomic analysis enlightens Agaricales lifestyle evolution and increasing peroxidase diversity. Mol Biol Evol. 38:1428–1446. 10.1093/molbev/msaa301 PMC8480192 33211093

[evad136-B60] Sánchez-García M , et al 2020. Fruiting body form, not nutritional mode, is the major driver of diversification in mushroom-forming fungi. Proc Natl Acad Sci U S A. 117:32528–32534. 33257574 10.1073/pnas.1922539117 PMC7768725

[evad136-B61] Sanderson MJ . 2003. R8s: inferring absolute rates of molecular evolution and divergence times in the absence of a molecular clock. Bioinformatics 19:301–302. 12538260 10.1093/bioinformatics/19.2.301

[evad136-B62] Simão FA , Waterhouse RM, Ioannidis P, Kriventseva EV, Zdobnov EM. 2015. BUSCO: assessing genome assembly and annotation completeness with single-copy orthologs. Bioinformatics 31:3210–3212. 26059717 10.1093/bioinformatics/btv351

[evad136-B63] Sipos G , et al 2017. Genome expansion and lineage-specific genetic innovations in the forest pathogenic fungi *Armillaria*. Nat Ecol Evol. 1:1931–1941. 29085064 10.1038/s41559-017-0347-8

[evad136-B64] Sønstebø JH , et al 2022. Population genomics of a forest fungus reveals high gene flow and climate adaptation signatures. Mol Ecol. 31:1963–1979. 35076968 10.1111/mec.16369

[evad136-B65] Stamatakis A . 2014. RAxML version 8: a tool for phylogenetic analysis and post-analysis of large phylogenies. Bioinformatics 30:1312–1313. 24451623 10.1093/bioinformatics/btu033 PMC3998144

[evad136-B66] Stanke M , Waack S. 2003. Gene prediction with a hidden Markov model and a new intron submodel. Bioinformatics 19:ii215–ii225. 14534192 10.1093/bioinformatics/btg1080

[evad136-B67] Steidinger BS , et al 2019. Climatic controls of decomposition drive the global biogeography of forest-tree symbioses. Nature 569:404–408. 31092941 10.1038/s41586-019-1128-0

[evad136-B68] Sun YF , et al 2022. Phylogenomics and comparative genomics highlight specific genetic features in *Ganoderma* species. J Fungi (Basel). 8:311. 35330313 10.3390/jof8030311 PMC8955403

[evad136-B69] Tang Y , Horikoshi M, Li WX. 2016. Ggfortify: unified interface to visualize statistical results of popular R packages. R J. 8:474–485.

[evad136-B70] Tedersoo L , Smith ME. 2013. Lineages of ectomycorrhizal fungi revisited: foraging strategies and novel lineages revealed by sequences from belowground. Fungal Biol Rev. 27:83–99.

[evad136-B71] Tedersoo L , Suvi T, Beaver K, Saar I. 2007. Ectomycorrhizas of *Coltricia* and *Coltriciella* (Hymenochaetales, Basidiomycota) on Caesalpiniaceae, Dipterocarpaceae and Myrtaceae in Seychelles. Mycol Prog. 6:101–107.

[evad136-B72] Thakur VK , Thakur MK. 2014. Processing and characterization of natural cellulose fibers/thermoset polymer composites. Carbohyd Polym. 109:102–117. 10.1016/j.carbpol.2014.03.039 24815407

[evad136-B73] Varga T , et al 2019. Megaphylogeny resolves global patterns of mushroom evolution. Nat Ecol Evol. 3:668–678. 30886374 10.1038/s41559-019-0834-1 PMC6443077

[evad136-B74] Vurture GW , et al 2017. Genomescope: fast reference-free genome profiling from short reads. Bioinformatics 33:2202–2204. 28369201 10.1093/bioinformatics/btx153 PMC5870704

[evad136-B75] Wang K , Kirk PM, Yao YJ. 2019. The development trends in taxonomy, with a special reference to fungi. J Syst Evol. 58:406–412.

[evad136-B76] Wang XQ , Ran JH. 2014. Evolution and biogeography of gymnosperms. Mol Phylogenet Evol. 75:24–40. 24565948 10.1016/j.ympev.2014.02.005

[evad136-B77] Whitham TG , et al 2008. Extending genomics to natural communities and ecosystems. Science 320:492–495. 18436780 10.1126/science.1153918

[evad136-B78] Wickham H . 2011. Ggplot2. Wiley Interdiscip Rev Comput Stat. 3:180–185.

[evad136-B79] Won H , Renner SS. 2006. Dating dispersal and radiation in the gymnosperm *Gnetum* (Gnetales)—clock calibration when outgroup relationships are uncertain. Syst Biol. 55:610–622. 16969937 10.1080/10635150600812619

[evad136-B80] Wu F , et al 2019. Resource diversity of Chinese macrofungi: edible, medicinal and poisonous species. Fungal Divers. 98:1–76.

[evad136-B81] Wu G , et al 2022c. Evolutionary innovations through gain and loss of genes in the ectomycorrhizal Boletales. New Phytol. 233:1383–1400. 34767630 10.1111/nph.17858

[evad136-B82] Wu F , Man XW, Tohtirjap A, Dai YC. 2022a. A comparison of polypore funga and species composition in forest ecosystems of China, North America, and Europe. For Ecosyst. 9:100051.

[evad136-B83] Wu F , Zhou LW, Vlasák J, Dai YC. 2022b. Global diversity and systematics of Hymenochaetaceae with poroid hymenophore. Fungal Divers. 113:1–192.

[evad136-B84] Zhang C , et al 2021. Discriminating symbiosis and immunity signals by receptor competition in rice. Proc Natl Acad Sci U S A. 118:e2023738118. 10.1073/pnas.2023738118 PMC8072404 33853950

[evad136-B85] Zhao H , Liu X-Y, Wu F. 2022a. The complete mitochondrial genome of *Porodaedalea mongolica* (Hymenochaetaceae, Basidiomycota). Mitochondrial DNA B Resour. 7:913–915. 35692659 10.1080/23802359.2022.2078677 PMC9176326

[evad136-B86] Zhao H , Zhou M, Liu XY, Wu F, Dai YC. 2022b. Phylogeny, divergence time estimation and biogeography of the genus *Onnia* (Basidiomycota, Hymenochaetaceae). Front Microbiol. 13:907961. 10.3389/fmicb.2022.907961 PMC9301299 35875515

[evad136-B87] Zimin AV , et al 2013. The MaSuRCA genome assembler. Bioinformatics 29:2669–2677. 23990416 10.1093/bioinformatics/btt476 PMC3799473

