# Supplementary material for: Insights into the Ecological Diversification of the Hymenochaetales based on Comparative Genomics and Phylogenomics With an Emphasis on Coltricia
Source: Genome Biol Evol. 2023 Jul 27;15(8):evad136. doi: 10.1093/gbe/evad136 (PMC10410303; doi:10.1093/gbe/evad136)
Supplement: evad136_Supplementary_Data [file evad136_supplementary_data.zip › Supplemenarty Figures.docx]

**Figure S1.** Distribution of the numbers of secreted auxiliary activities (a), secreted carbohydrate binding modules (b), secreted carbohydrate esterases (c), secreted glycoside hydrolases (d), secreted glycosyl transferases (e), and secreted polysaccharide lyases (f) among secreted carbohydrate-active enzymes explained by ecological groups. Three different colors in plots highlight three different ecological groups, respectively. * and ** indicate significantly different ectomycorrhizal species compared with ecological groups (*P* < 0.05 and *P* < 0.01, respectively).


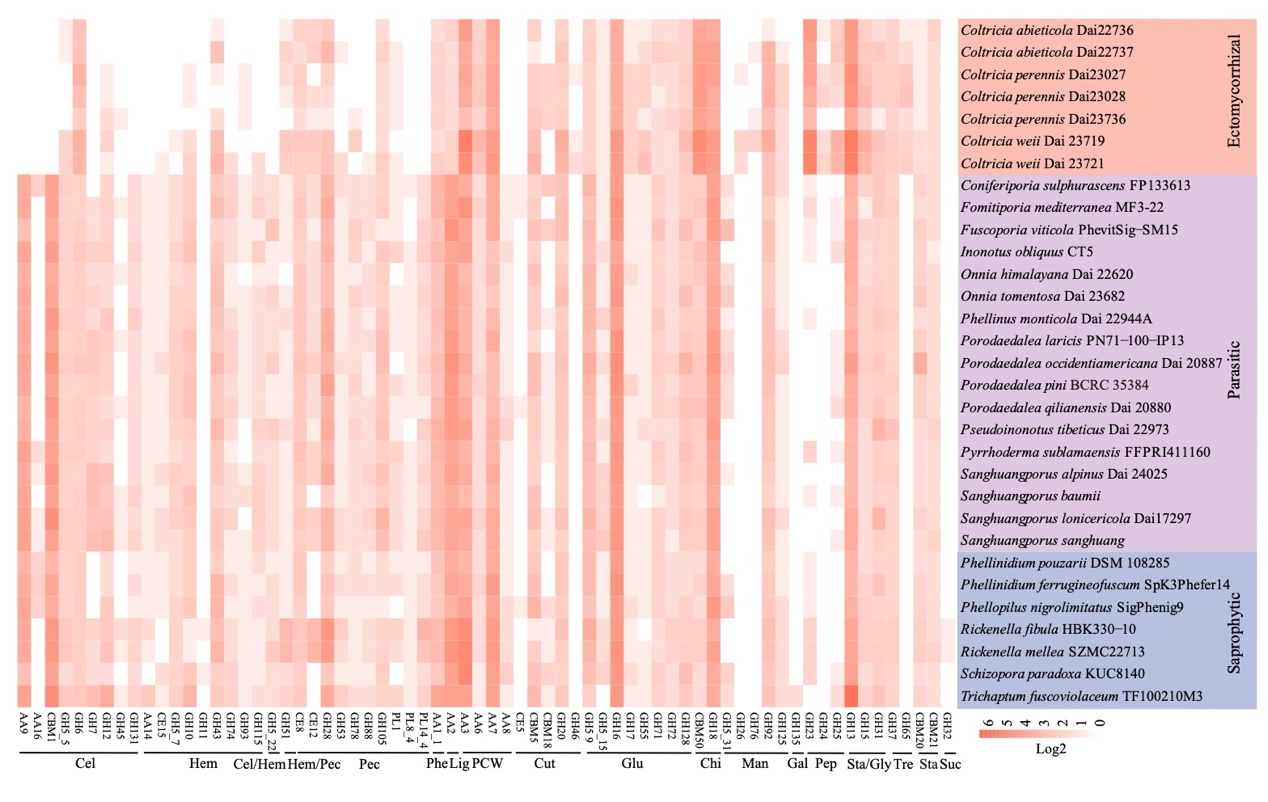


**Figure S2.** Distribution of classical total CAZyme domains in different ecological groups in the identified CAZyme genes corresponding to nine auxiliary activities (AAs), six carbohydrate-binding module (CBMs), four carbohydrate esterase (CEs), 46 glycoside hydrolase (GHs), and three polysaccharide lyase (PLs) families. Three different colors highlight three different ecological groups, respectively. Abbreviation: Cel - cellulose, Hem - hemicellulose, Pec - pectin, Phe - phenols, Lig - lignin, PCW - partial plant cell-wall degradation, Cut - cutin, Glu - glucan, Chi - chitin, Man - mannan, Gal - galactosaminogalactan, Pep - peptidoglycan, Sta - starch, Gly - glycogen, Tre - trehalose, Suc - sucrose.


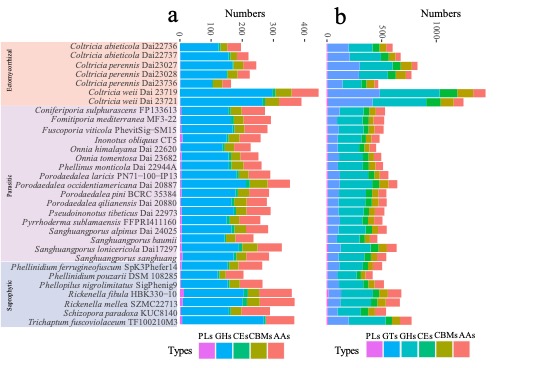


**Figure S3.** Distribution of the classical secreted (a) and total (b) CAZyme families.

**Figure S4.** Distribution of the numbers of secreted CAZyme families involved in the degradation of cellulose and lignin explained by host trees: a - AA9, b - AA16, c - CBM1, d - GH5_5, e - GH6, f - GH7, g - GH12, h - GH45, i - GH131 family and l - AA2 families. Three different colors highlight three different host groups in plots, respectively.

**Figure S5.** Principal component analyses (PCA) of host plants and ecological group based on CAZyme domains **(a)**, host plants and ecological group based on secreted CAZyme domains **(b)**, host plants and ecological groups based on total CAZyme domains. An: angiosperms, Br: bryophyte, Gy: gymnosperms.
